# Relaxation Dynamics of Semiflexible Fractal Macromolecules

**DOI:** 10.3390/polym8070263

**Published:** 2016-07-15

**Authors:** Jonas Mielke, Maxim Dolgushev

**Affiliations:** 1 Institute of Physics, University of Freiburg, Hermann-Herder-Str. 3, 79104 Freiburg, Germany; jonas.mielke@saturn.uni-freiburg.de; 2 Institut Charles Sadron, Université de Strasbourg & CNRS, 23 rue du Loess, 67034 Strasbourg Cedex, France

**Keywords:** hyperbranched polymers, semiflexibility, fractals, pseudo-dendrimers, mechanical relaxation, eigenmodes

## Abstract

We study the dynamics of semiflexible hyperbranched macromolecules having only dendritic units and no linear spacers, while the structure of these macromolecules is modeled through T-fractals. We construct a full set of eigenmodes of the dynamical matrix, which couples the set of Langevin equations. Based on the ensuing relaxation spectra, we analyze the mechanical relaxation moduli. The fractal character of the macromolecules reveals itself in the storage and loss moduli in the intermediate region of frequencies through scaling, whereas at higher frequencies, we observe the locally-dendritic structure that is more pronounced for higher stiffness.

## 1. Introduction

Macromolecular systems with a high amount of branching units continue to attract high attention [1,2,3,4,5,6,7]. Typical representatives of such systems are hyperbranched polymers and dendrimers. While dendrimers possess a perfect layered topology, hyperbranched polymers can have a high structural variety. Moreover, hyperbranched polymers represent a very broad class of macromolecular structures, given also that there is a possibility [4,8] to tune their degree of branching (the degree of branching reflects the ratio between branching points and linear spacers [9]). However, a characterization based only on the degree of branching is rather superficial, because it does not carry information about the distribution of the branching points, i.e., the monomer connectivity [10,11,12,13,14]. A prominent example in this respect is provided by pseudo-dendrimers that possess the same degree of branching as dendrimers, but distinct physical properties [14,15]. Here, we study a system with a fractal connectivity, which, as for dendrimers and pseudo-dendrimers, does not possess linear spacers.

**Figure 1:**
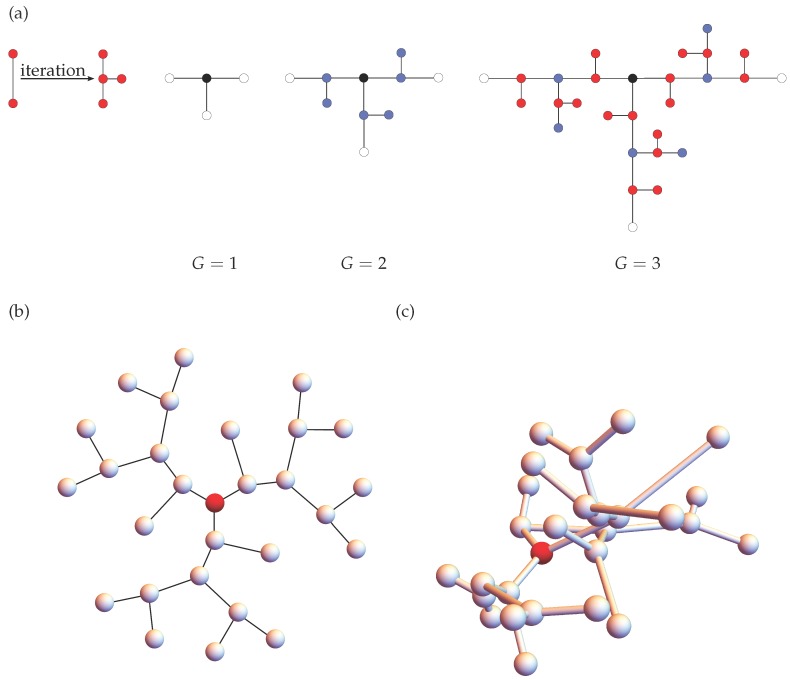
(**a**) Iterative construction of a T-fractal up to generation G=3. The beads added in the second and in the third iteration steps are colored by blue and red, respectively; (**b**) A two-dimensional representation of a T-fractal of generation G=3, and (**c**) its random configuration in three-dimensional space.

Let us now briefly introduce the fractal system, the so-called “T-fractal”, on which we focus here. Belonging to the class of exactly decimable fractals, T-fractals enjoy a constant theoretical attention [16,17,18,19,20,21,22,23,24,25,26]. Figure 1a illustrates the iterative construction of a T-fractal up to the third generation: In every iteration step, each bond of a T-fractal is substituted through three other bonds. We note that Figure 1a sketches only the topology of a T-fractal. In fact, we are considering here T-fractals with homogeneous branching units, so that their topology resembles rather that of pseudo-dendrimers, as sketched in Figure 1b. Moreover, in three dimensions, the structures will have typically different conformations; Figure 1c exemplifies a randomly-taken conformation from a multivariate Gaussian distribution related to the macromolecule. We note that in contrast to dendrimers or to dendrons (dendritic wedges), T-fractals contain beads, which do not branch out further. The absence of the corresponding sub-wedges can allow T-fractals to reach quite high generations, while dendrimers and dendrons, which experience problems related to the space filling, are seriously limited in their growth.

The theoretical description of a polymer’s dynamics requires a suitable mathematical model. In order to find the relationship between the dynamics of a macromolecule and its topology, one can use in a first approach the model of generalized Gaussian structures (GGS) [27], which originates from the Rouse model [28]. However, the GGS model does not include the excluded volume and the restrictions of the bond angles of macromolecules. An improved description of the polymer’s dynamics is achieved by introducing local semiflexibility in the GGS model, which turns out to be very important for dendritic structures [29,30]. Semiflexibility was first introduced to the dynamics of discrete chains by Bixon and Zwanzig [31]; later, it was included to the description of other macromolecular architectures [32,33,34,35,36,37,38,39,40,41,42]. This work employs the framework of semiflexible treelike polymers (STP) [35], which allows one to study arbitrary treelike architectures and to obtain many results in closed form. In particular, the STP framework allows us to determine in this work a complete set of eigenmodes of semiflexible T-fractals, following the procedure put forward by Cai and Chen for fully-flexible dendrimers [43] that has been recently extended to semiflexible structures, namely dendrimers [39] and Vicsek fractals [40]. This procedure reduces the numerical effort and gives an intuitive sense to the structure of eigenmodes and of the corresponding relaxation spectra. These results allow us to consider here the mechanical relaxation forms of very large macromolecules and to understand their dynamical behavior in depth.

The outline of the paper is as follows: Section 2 recalls briefly the methods, while our results are presented in Section 3. In particular, Section 3 starts with the description of the elements of the dynamical matrix, for which we then construct a complete set of eigenmodes for semiflexible T-fractals and the corresponding reduced matrices; afterwards, we discuss the eigenvalue spectra and corresponding mechanical relaxation moduli. Finally, Section 4 summarizes our conclusions. Appendix A. contains a general iterative procedure for the construction of reduced dynamical matrices.

## 2. Methods

In this section, the model of semiflexible treelike polymers (STP) is briefly recalled; details of the STP model can be found in [35].

In the STP-model, polymer structures are described by beads, located at the positions $r_{i}$ (i=1,...,N), that are connected by bonds with the bond vector:

(1) $$\begin{array}{c} d_{a}=r_{i}-r_{j}=\sum_{n}{\left(G^{T}\right)}_{an}r_{n} \end{array}$$

In Equation (1) the incidence matrix G known from graph theory is used.

Considering the easiest case, the so-called GGS-model [27] that extends the Rouse model to arbitrary architectures, one obtains a purely harmonic potential $V_{\mathrm{GGS}}$ that is diagonal in the variables representing the bonds:

(2) $$\begin{array}{c} V_{\mathrm{GGS}}\left(\left\{d_{a}\right\}\right)=\frac{K}{2}\sum_{a}d_{a}^{2} \end{array}$$

$K=\frac{3k_{B}T}{\ell^{2}}$ denotes the spring constant, where $\ell^{2}$ is the mean squared length of the bonds, *T* the temperature and $k_{B}$ the Boltzmann constant.

Neither the volume of the monomers nor restrictions on the bond angles are considered in the GGS-model. A first approximation taking into account these restrictions leads to a correlation of successive bonds. Introducing semiflexibility in the GGS-model by imposing geometrical restrictions for the bonds’ orientations results in a generalized potential:

(3) $$\begin{array}{c} V_{\mathrm{STP}}\left(\left\{d_{a}\right\}\right)=\frac{K}{2}\sum_{a,b}W_{ab}d_{a}\cdot d_{b} \end{array}$$

The matrix W contains the information about the correlation between the different bonds. The structure of potential (3) can be obtained based on maximum entropy methods [35,44] or by construction of the covariance matrix (consisting of the mean values $\left\{\langle d_{a}\cdot d_{b}\rangle \right\}$) of the respective Boltzmann distribution $\frac{1}{Z}\exp\left(-\frac{V_{\mathrm{STP}}}{k_{B}T}\right)$. With this, one has $\langle d_{a}\cdot d_{b}\rangle =\ell^{2}{\left(W^{-1}\right)}_{ab}$ for Gaussian distributed bonds $\left\{d_{a}\right\}$. In order to obtain the matrix W, by inverting $W^{-1}$, the following physically-plausible choices for $\langle d_{a}\cdot d_{b}\rangle$ are made:

- The mean squared length of the bonds is fixed $\langle d_{a}\cdot d_{b}\rangle =\ell^{2}$.
- For adjacent bonds *a* and *b*, directly connected over a bead *i*, $\langle d_{a}\cdot d_{b}\rangle =\pm \ell^{2}q_{i}$ holds. The sign is determined by the relative orientation of the bonds. The positive sign describes the case of head-to-tail orientation of *a* and *b*; otherwise, the minus sign is obtained. The common stiffness parameter related to *a* and *b* is denoted by $q_{i}$.
- Due to the freely-rotating condition imposed on non-adjacent bonds *a* and *c* (connected over the unique path $\left(b_{1},...,b_{k}\right)$), one obtains $\langle d_{a}\cdot d_{c}\rangle =\langle d_{a}\cdot d_{b_{1}}\rangle \langle d_{b_{1}}\cdot d_{b_{2}}\rangle \cdots \langle d_{b_{k}}\cdot d_{c}\rangle \ell^{-2k}$. For *linear chains*, this restriction under the continuous chain limit ℓ→0 and $q_{i}\to 1$ leads to the definition of the persistence length $L_{p}$; see Equation (3.15) of [44]. However, for branched structures, no smooth curve description due to the branching points is possible.

In the limit $q_{i}\to 0$, W resembles the identity matrix, in which case Equations (2) and (3) coincide. For a branching point of functionality (i.e., number of NN) $f_{i}$, there is a restriction concerning the upper limit of the stiffness parameter $q_{i}$, $q_{i}\leq \frac{1}{f_{i}-1}$; see [45]. This restriction comes from the observation of $f_{i}$ rays emanating from the same origin, for which in three-dimensional space the sum of cosines of the angles between them ($f_{i}$ rays lead to $f_{i}\left(f_{i}-1\right)/2$ angles) is restricted by $-f_{i}/2$ from below [45]. A detailed presentation of the explicit matrix elements of W, taking into account the above conditions, can be found in [35].

Within the framework of the STP-model, the following set of Langevin equations describes the dynamics of the polymer:

(4) $$\begin{array}{c} \zeta \frac{\partial }{\partial t}r_{i}\left(t\right)+\frac{\partial }{\partial r_{i}}V_{\mathrm{STP}}\left(\left\{r_{k}\right\}\right)=g_{i}\left(t\right) \end{array}$$

*ζ* denotes the friction coefficient of a bead. The stochastic Gaussian force acting on the *i*-th bead $g_{i}$ has the properties $\langle g_{i}\left(t\right)\rangle =0$ and $\langle g_{i\alpha }\left(t\right)g_{j\beta }\left(t^{\prime }\right)\rangle =2k_{B}T\zeta \delta_{i,j}\delta_{\alpha ,\beta }\delta \left(t-t^{\prime }\right)$, where *α* and *β* denote the three spatial directions x,y and *z*.

The system of Langevin equations (4) requires that the potential $V_{\mathrm{STP}}$ is expressed in terms of the position variables $\left\{r_{i}\right\}$. Combining (1) and (3), one obtains:

(5) $$\begin{array}{cc} V_{\mathrm{STP}}\left(\left\{r_{n}\right\}\right) & =\frac{K}{2}\sum_{i,j}{\left(\underset{A^{\mathrm{STP}}}{\underset{︸}{{\mathrm{GWG}}^{T}}}\right)}_{ij}r_{i}\cdot r_{j}=\frac{K}{2}\sum_{i,j}{\left(A^{\mathrm{STP}}\right)}_{ij}r_{i}\cdot r_{j} \end{array}$$

Referring to one picked bead *i* (with functionality $f_{i}$ and stiffness parameter $q_{i}$), there are three types of non-vanishing matrix elements of $A^{\mathrm{STP}}$; namely, the diagonal element $A_{ii}^{\mathrm{STP}}$, the nearest neighbor (NN) elements $A_{ii_{k}}^{\mathrm{STP}}$, where $i_{k}$ denotes the NN of *i*, and the next nearest neighbor (NNN) elements $A_{ii_{ks}}^{\mathrm{STP}}$, where $i_{ks}$ denotes the NN of $i_{k}$ excluding bead *i*. In the following, $f_{i_{k}}$ and $q_{i_{k}}$ denote the functionality and the stiffness parameter associated with bead $i_{k}$, respectively. As has been shown in [35], the analytical expressions for these three non-vanishing matrix elements are:

(6) $$\begin{array}{cc} A_{ii}^{\mathrm{STP}}= & \frac{f_{i}}{1-\left(f_{i}-1\right)q_{i}}+\sum_{i_{k}}\frac{\left(f_{i_{k}}-1\right)q_{i_{k}}^{2}}{1-\left(f_{i_{k}}-2\right)q_{i_{k}}-\left(f_{i_{k}}-1\right)q_{i_{k}}^{2}} \end{array}$$

(7) $$\begin{array}{c} A_{ii_{k}}^{\mathrm{STP}}=-\frac{1-\left(f_{i}-1\right)\left(f_{i_{k}}-1\right)q_{i}q_{i_{k}}}{\left(1-\left(f_{i}-1\right)q_{i}\right)\left(1-\left(f_{i_{k}}-1\right)q_{i_{k}}\right)} \end{array}$$

and:

(8) $$\begin{array}{c} A_{ii_{ks}}^{\mathrm{STP}}=\frac{q_{i_{k}}}{1-\left(f_{i_{k}}-2\right)q_{i_{k}}-\left(f_{i_{k}}-1\right)q_{i_{k}}^{2}} \end{array}$$

We note that if any of the beads (*i* or $i_{k}$) has functionality one, then the corresponding stiffness parameter ($q_{i}$ or $q_{i_{k}}$) in Equations (6) and (7) is multiplied by a zero (e.g., by $f_{i}-1$ or by $f_{i_{k}}-1$). Such beads do not connect any pair of bonds, and hence, Equations (6) and (7) automatically account for this fact by refusing an input of the corresponding stiffness parameters. Equation (8) appears only for the situation where bead $i_{k}$ connects at least two other beads; therefore, in Equation (8), $f_{i_{k}}\geq 2$. T-fractals have only beads of functionality one or three. Here, we consider a homogeneous situation by having the same stiffness parameter *q* for all beads of functionality three.

The isotropy of the model leads for each bead to a decoupling of the three spatial coordinates. Hence, the equation describing the dynamics, say, of the *i*-th bead’s *x*-component, reads:

(9) $$\begin{array}{c} \zeta \frac{\partial }{\partial t}x_{i}\left(t\right)+K\sum_{j=1}^{N}A_{ij}^{\mathrm{STP}}x_{j}\left(t\right)=g_{ix}\left(t\right) \end{array}$$

Within the STP-model, many dynamical properties of a polymer are determined to a large extent by the eigenvalues of $A^{\mathrm{STP}}$. For $A^{\mathrm{STP}}$, the sum of all elements in any row or in any column vanishes. This leads to the eigenvalue $\lambda_{1}=0$ whose corresponding eigenvector describes the translation of the complete macromolecule. The other, non-vanishing eigenvalues $\left\{\lambda_{2},\lambda_{3},...\right\}$ are sufficient to describe various dynamical properties, such as the mechanical relaxation behavior. In order to investigate this behavior, one considers the response to the harmonic strain, represented through external mechanical forces that oscillate with the frequency *ω*. The response function to this harmonic strain is the complex shear modulus $G^{*}\left(\omega \right)=G^{\prime }\left(\omega \right)+iG^{\prime \prime }\left(\omega \right)$ (see [46]), consisting of the storage modulus $G^{\prime }\left(\omega \right)$ and the loss modulus $G^{\prime \prime }\left(\omega \right)$. The analytical expressions of the two moduli expressed in the reduced variables that are obtained by dividing the moduli by $\nu k_{B}T$ are given by [27,36]:

(10) $$\begin{array}{c} \left[G^{\prime }\left(\omega \right)\right]=\frac{G^{\prime }\left(\omega \right)}{\nu k_{B}T}=\frac{1}{N}\sum_{k=2}^{N}\frac{{\left(\frac{\omega \tau_{0}}{2\lambda_{k}}\right)}^{2}}{1+{\left(\frac{\omega \tau_{0}}{2\lambda_{k}}\right)}^{2}} \end{array}$$

and:

(11) $$\begin{array}{c} \left[G^{\prime \prime }\left(\omega \right)\right]=\frac{G^{\prime \prime }\left(\omega \right)}{\nu k_{B}T}=\frac{1}{N}\sum_{k=2}^{N}\frac{\frac{\omega \tau_{0}}{2\lambda_{k}}}{1+{\left(\frac{\omega \tau_{0}}{2\lambda_{k}}\right)}^{2}} \end{array}$$

One should note that in Equations (10) and (11), only the non-vanishing eigenvalues $\left\{\lambda_{k}\right\}$ contribute.

## 3. Results and Discussion

### 3.1. Dynamical Matrix of T-fractals

As we discussed in Section 2, the dynamics of macromolecules is modeled through a set of Langevin equations, which are coupled through the dynamical matrix $A^{\mathrm{STP}}$. Equations (6)–(8) of Section 2 allow the full determination of the elements of the N×N matrix $A^{\mathrm{STP}}$, where *N* is the number of beads of a T-fractal that for generation *G* reads:

(12) $$\begin{array}{c} N\left(G\right)=3^{G}+1 \end{array}$$

Below, we discuss all possible non-vanishing entries of $A^{\mathrm{STP}}$ occurring for T-fractals. Namely, there are three classes of entries: diagonal elements (*μ*), those related to nearest neighboring (NN) beads (*ν*) and those to the next-nearest neighboring (NNN) ones (*ρ*). All of these elements depend on the stiffness parameter *q*, which reflects the bonds’ orientations; see Section 2 for details.

**Figure 2:**
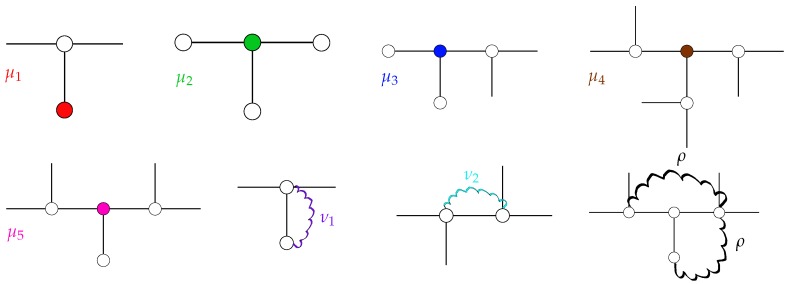
Schematic representation of the non-vanishing elements of matrix $A^{\mathrm{STP}}$. For diagonal elements ($\mu_{i}$), the corresponding beads are highlighted by color. For off-diagonal elements ($\nu_{i}$ and *ρ*), the corresponding interactions are indicated through wavy lines.

Each diagonal element corresponds to a bead. Therefore, the diagonal elements depend on the beads’ topological position within the T-fractal. The topology of T-fractals results in five different diagonal elements, as shown in Figure 2.

1. Terminal beads with functionality (i.e., number of NN) f=1 have exactly one internal NN with f=3. Hence, one obtains the matrix element:
  (13) $$\mu_{1}=1+\frac{2q^{2}}{1-q-2q^{2}}$$
2. For T-fractals of generation G=1, the single internal bead is directly connected to three terminal beads. The corresponding matrix element is given by:
  (14) $$\mu_{2}=\frac{3}{1-2q}$$
3. An internal bead with two terminal and one internal NN is described by the diagonal element:
  (15) $$\mu_{3}=\frac{3}{1-2q}+\frac{2q^{2}}{1-q-2q^{2}}$$
4. The diagonal element $\mu_{4}$ corresponds to internal beads with three internal NN:
  (16) $$\mu_{4}=\frac{3}{1-2q}+3\frac{2q^{2}}{1-q-2q^{2}}$$
5. If an internal bead has two internal and one terminal NN, it is described by the diagonal element:
  (17) $$\begin{array}{c} \mu_{5}=\frac{3}{1-2q}+2\frac{2q^{2}}{1-q-2q^{2}} \end{array}$$

Besides the diagonal elements, there are two types of non-vanishing NN elements following from the analysis of NN pairs of beads, as depicted in Figure 2.

1. If one of the two considered beads is a terminal bead, we obtain the NN element:
  (18) $$\nu_{1}=-\frac{1}{1-2q}$$
2. Otherwise, two internal beads in NN position result in the matrix element:
  (19) $$\nu_{2}=-\frac{1+2q}{1-2q}$$

According to Equation (8) of Section 2, the NNN elements depend solely on the properties of the beads, which are common NN to the NNN pairs of beads. Since this common NN is inevitably an internal bead and given that all internal beads of a T-fractal have functionality f=3, all NNN elements are equal to:

(20) $$\rho =\frac{q}{1-q-2q^{2}}$$

### 3.2. Hierarchical Eigenmodes of T-fractals

**Figure 3:**
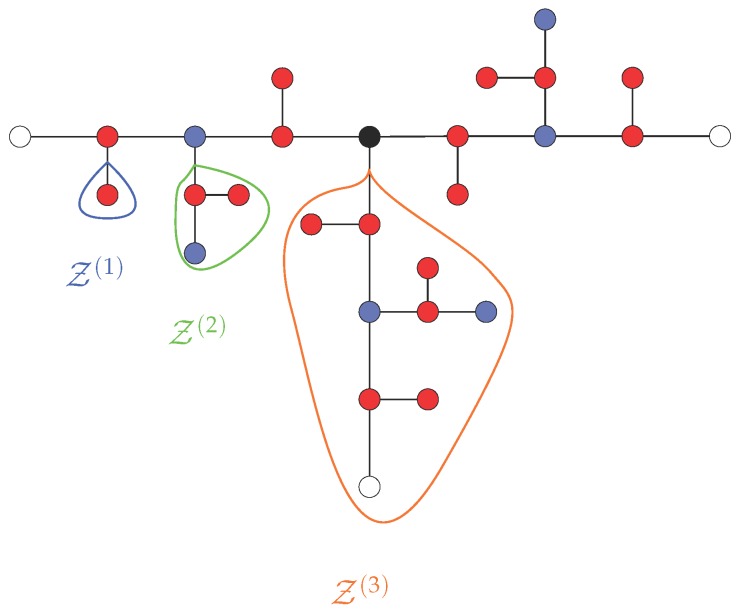
Branches $Z^{\left(G_{Z}\right)}$ of different branch generation $G_{Z}=1,2,3$ for a T-fractal of generation G=3.

In order to analyze the eigenmodes of the T-fractals, we concentrate on the homogeneous form of the set of Langevin equations introduced in Section 2; see Equation (9):

(21) $$\begin{array}{c} \tau_{0}{\dot{x}}_{i}+\sum_{j=1}^{N}A_{ij}^{\mathrm{STP}}x_{j}=0\;\forall i \end{array}$$

where $\tau_{0}=\zeta /K$ is the monomeric relaxation time. A complete numerical diagonalization of the N(G)×N(G) matrix $A^{\mathrm{STP}}$ allows one to determine the eigenmodes. Since N(G), Equation (12), grows exponentially, a numerical diagonalization is only possible for low generations of T-fractals. Using a complete set of eigenvectors of $A^{\mathrm{STP}}$ reduces the computational effort. Such a procedure was first introduced for fully flexible dendrimers of functionality f=3 [43] and later extended to arbitrary functionalities [47,48]; see also recent general results [49] for flexible dendritic structures. The works in [39,40] illustrate that an extension of the procedure is also applicable to semiflexible dendrimers and semiflexible Vicsek fractals. Here, we find (G+1) groups of eigenvectors for a T-fractal of generation *G*. Among them, the first *G* groups are based on the branches $Z^{\left(1\right)}$ to $Z^{\left(G\right)}$, whereas the (G+1)-th group involves the motion of all beads, including the central one (in the case of semiflexible dendrimers [39], the groups 1 to *G* represent the dynamics of dendrons of the generations 1 to *G*, and the (G+1)-th group involves the motion of all dendrimer’s beads). A branch $Z^{\left(G_{Z}\right)}$ of generation $G_{Z}=1$ consists of a single terminal bead. In general, branches are connected to the rest of the structure over one bead, as depicted in Figure 3, which is called the “starting” bead in the following.

The *n*-th group (n=1,...,G) of eigenvectors is characterized by the movements of pairs of the $Z^{\left(n\right)}$ branches, while the remaining part of the macromolecule stands immobile. Since a large part of the fractal is immobile, for a specific group of eigenvectors, many amplitudes $x_{j}$ in Equation (21) vanish.

**Figure 4:**
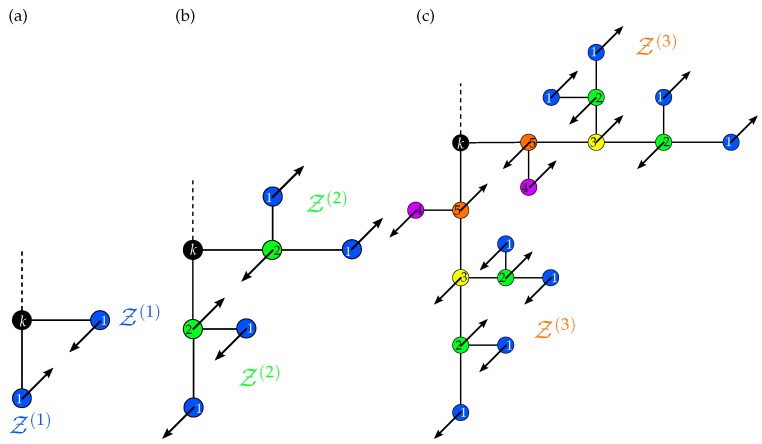
Examples for eigenmodes of the first (**a**), second (**b**) and third (**c**) group. The beads of a branch that move with the same amplitude have the same color. The numbers placed in the beads correspond to the variables $\left\{x_{i}\right\}$ used in Equations (23), (25) and (26). The beads *k* colored by black are the gluing beads.

The eigenvectors of the first group describe antiphase movements of two terminal beads (say, *i* and *j*) connected by a common NN (say, *k*). Each of these terminal beads represents a $Z^{\left(1\right)}$ branch, as depicted in Figure 4. The antiphase movement of the two beads *i* and *j* results in the relations:

(22) $$\begin{array}{c} x_{1}:=x_{i}=-x_{j}\;\text{and}\;x_{l}=0\;\text{for}\;\text{all}\;i\neq l\neq j \end{array}$$

for the amplitudes. Using Equations (13) and (20), one obtains the diagonal elements $A_{ii}^{\mathrm{STP}}=A_{jj}^{\mathrm{STP}}=\mu_{1}$ and the NNN elements $A_{ij}^{\mathrm{STP}}=A_{ji}^{\mathrm{STP}}=\rho$. With this, the set of Equations (21) reduces to the following non-trivial equation of motion:

(23) $$\begin{array}{c} -\tau_{0}{\dot{x}}_{1}=\mu_{1}x_{1}-\rho x_{1}=\left(\mu_{1}-\rho \right)x_{1} \end{array}$$

Since the chosen model is limited to NNN interactions, the matrix element, say, $A_{lm}^{\mathrm{STP}}$ of two beads *l* and *m*, connected by a path containing more than two bonds, vanishes. Hence, in case of immobile beads, it is necessary to discuss only the equations of motion of bead *k* and its immobile NN. In both cases, the sum in Equation (21) leads to zero, as a result of the antiphase movement of *i* and *j*. Measuring time in units of $\tau_{0}$ and making use of Equations (13) and (20), one can easily calculate from Equation (23) the eigenvalue corresponding to the first group:

(24) $$\begin{array}{c} \lambda^{\left(1\right)}=\mu_{1}-\rho =\frac{1}{1+q} \end{array}$$

The second group is related to the opposing movement of two $Z^{\left(2\right)}$ branches, which are connected through an immobile bead, say *k*; see Figure 4b. In the following, we call the bead *k* the “gluing” bead. The beads of one branch depicted in the same color in Figure 4b are arranged in a symmetric way with respect to the gluing bead. For these beads, the movement amplitudes are identical, whereas the beads of one branch move opposite of their symmetric counterparts in the second branch: the starting beads (NN to *k*) of the two branches perform an opposing movement, whereby the amplitudes $x_{2}$ and $-x_{2}$ have the same absolute value; for all other mobile beads, the absolute value of their amplitudes is $|x_{1}|$. Due to the opposing movement of the different groups of beads of the two branches, the sums in the equations of set (21) corresponding to the gluing bead *k* and its immobile NN vanish, so that the equations of motion of these two beads become trivial. Thus, considering the second group, the set of Equations (21) reduces to:

(25) $$\begin{array}{cc} -\tau_{0}{\dot{x}}_{1} & =\mu_{1}x_{1}+\nu_{1}x_{2}+\rho x_{1}=\left(\mu_{1}+\rho \right)x_{1}+\nu_{1}x_{1}\\ -\tau_{0}{\dot{x}}_{2} & =\mu_{3}x_{2}+2\nu_{1}x_{1}-\rho x_{2}=\left(\mu_{3}-\rho \right)x_{2}+2\nu_{1}x_{1} \end{array}$$

This set leads to two eigenvalues, $\lambda_{2}^{\left(1\right)}$ and $\lambda_{2}^{\left(2\right)}$, for the second group.

The third group describes the opposing movement of two $Z^{\left(3\right)}$ branches, whose starting beads are connected to a common immobile gluing bead, say *k*. Again, as for the second group, the equations of motion of *k* and its immobile NN are trivial, and the amplitudes of beads arranged in a symmetric way with respect to the gluing bead are identical. Thus, dealing with $Z^{\left(3\right)}$ branches, one has to take into account five groups of beads that are symmetric with respect to the gluing bead. Consequently, one needs a set of five independent variables $\left(x_{1},x_{2},...,x_{5}\right)$ to determine the eigenvalues and eigenvectors (the corresponding five groups of beads are highlighted by different colors in Figure 4c). Hence, the set of non-trivial equations of motion of the third group reads:

(26) $$\begin{array}{cc} -\tau_{0}{\dot{x}}_{1} & =\left(\rho +\mu_{1}\right)x_{1}+\nu_{1}x_{2}+\rho x_{3}\\ -\tau_{0}{\dot{x}}_{2} & =2\nu_{1}x_{1}+\left(\mu_{3}+\rho \right)x_{2}+\nu_{2}x_{3}+\rho x_{5}\\ -\tau_{0}{\dot{x}}_{3} & =4\rho x_{1}+2\nu_{2}x_{2}+\mu_{4}x_{3}+\rho x_{4}+\nu_{2}x_{5}\\ -\tau_{0}{\dot{x}}_{4} & =\rho x_{3}+\mu_{1}x_{4}+\nu_{1}x_{5}\\ -\tau_{0}{\dot{x}}_{5} & =2\rho x_{2}+\nu_{2}x_{3}+\nu_{1}x_{4}+\left(\mu_{5}-\rho \right)x_{5} \end{array}$$

Based on set (26), the third group yields five eigenvalues $\lambda_{3}^{\left(1\right)}$, $\lambda_{3}^{\left(2\right)}$, $\lambda_{3}^{\left(3\right)}$, $\lambda_{3}^{\left(4\right)}$ and $\lambda_{3}^{\left(5\right)}$.

**Figure 5:**
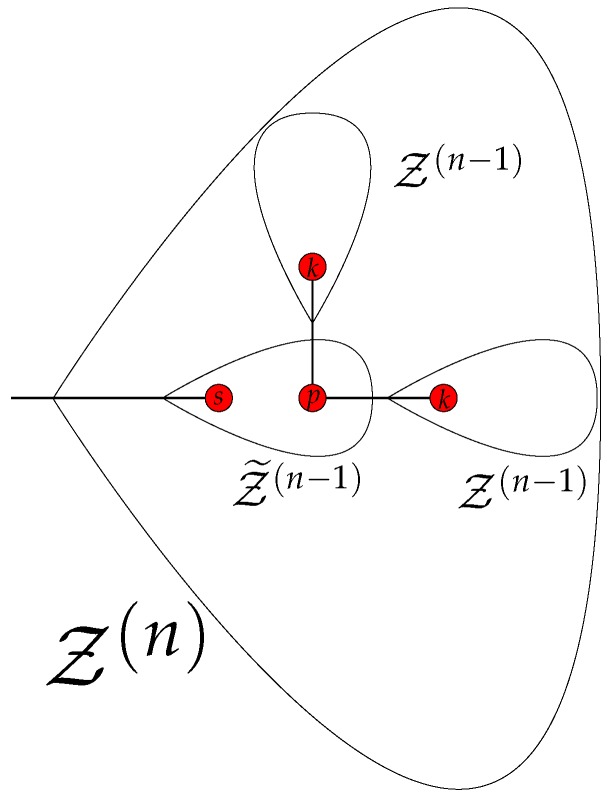
Iterative construction of a $Z^{\left(n\right)}$ branch consisting of three smaller branches; see the text for details.

Generally, the *n*-th group (with n≤G) describes motions of two $Z^{\left(n\right)}$ branches directly connected by a common gluing bead. The two branches perform an opposing movement, such that beads of one branch arranged symmetrically with respect to the gluing bead move with identical amplitudes and in antiphase to their counterparts of the second branch. Since the model considers only interactions up to NNN, the equations of motion for all immobile beads (including the gluing bead) are trivial. Therefore, the number of independent variables $\left\{x_{l}\right\}$ and, thus, the number of non-trivial equations of motion is determined by the number of groups of beads symmetric with respect to the gluing bead of one branch. The iterative construction of the branch $Z^{\left(n\right)}$, depicted in Figure 5, is helpful for the evaluation of the number of independent variables F(n) in the group *n*.

In the first step, a $Z^{\left(n\right)}$ branch gets decomposed into three branches (two of type $Z^{\left(n-1\right)}$ and one of ${\tilde{Z}}^{\left(n-1\right)}$). The two $Z^{\left(n-1\right)}$ branches have a similar structure as $Z^{\left(n\right)}$. All beads of both $Z^{\left(n-1\right)}$ branches, which are symmetric with respect to their mutual gluing bead, move inside the mobile branch $Z^{\left(n\right)}$ with the same amplitude and direction; see, e.g., Figure 4c for the motion of two branches $Z^{\left(2\right)}$ inside a mobile branch $Z^{\left(3\right)}$. Therefore, the contribution of the two $Z^{\left(n-1\right)}$ branches to F(n) is given by F(n-1).

**Figure 6:**
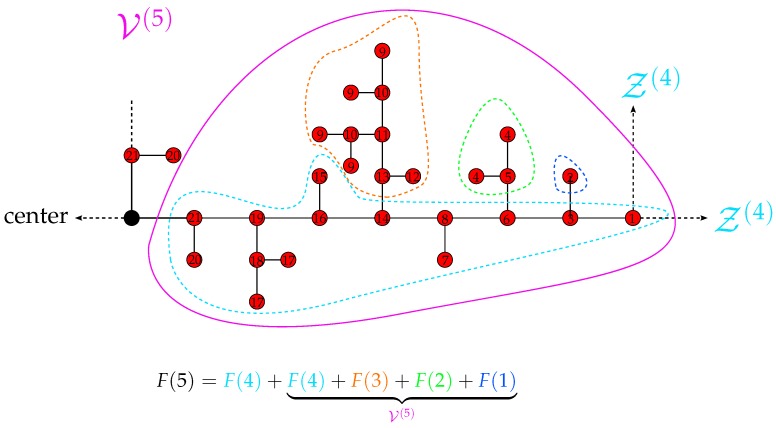
Composition of the number of independent variables F(5) for the fifth group; see the text for details.

In order to determine the remaining contribution $V^{\left(n\right)}\equiv F\left(n\right)-F\left(n-1\right)$ of the ${\tilde{Z}}^{\left(n-1\right)}$ branch, it is instructive to look at Figure 6 exemplifying the case n=5. The important observation is that for the ${\tilde{Z}}^{\left(n-1\right)}$ branch, the most distant beads from the center (beads labeled by 1, 2, 4 and 9 in Figure 6) have different functionalities, f=3 and f=1 (for $Z^{\left(n-1\right)}$, they are all of functionality f=1). This symmetry breaking leads to an increase of independent variables, since the branch $Z^{\left(n-2\right)}$ (beads having amplitudes numbered from 9 to 13) inside ${\tilde{Z}}^{\left(n-1\right)}$ does not have a symmetric counterpart and contributes solely to $V^{\left(n\right)}$. The same happens with the smaller branches $Z^{\left(n-3\right)},\cdots ,Z^{\left(1\right)}$; see Figure 6 for n=5. The branches $Z^{\left(n-2\right)},\cdots ,Z^{\left(1\right)}$ contribute to $V^{\left(n\right)}$ by F(n-2),⋯,F(1), respectively. All other beads inside the ${\tilde{Z}}^{\left(n-1\right)}$ branch move with amplitudes distinct from those of the separated $Z^{\left(n-2\right)},\cdots ,Z^{\left(1\right)}$ branches (whereas among them, the beads symmetric with respect to the gluing bead have the same amplitude) and contribute to $V^{\left(n\right)}$ by F(n-1). Summarizing, one finds:

(27) $$\begin{array}{c} V^{\left(n\right)}=\sum_{i=1}^{n-1}F\left(i\right) \end{array}$$

Using this result and accounting for the contribution of the two $Z^{\left(n-1\right)}$ branches inside the mobile $Z^{\left(n\right)}$ branch, it is straightforward to determine the number of independent variables F(n) for the *n*-th group:

(28) $$\begin{array}{c} F\left(n\right)=F\left(n-1\right)+V^{\left(n\right)}=F\left(n-1\right)+\sum_{i=1}^{n-1}F\left(i\right) \end{array}$$

In contrast to the *n*-th group (n≤G), the (G+1)-th group describes the case in which all beads of the T-fractal move (three $Z^{\left(G\right)}$ branches and the central bead). We note that the beads of the $Z^{\left(G\right)}$ branches, which are symmetric with respect to the central bead, have the same amplitude and phase. Therefore, the three $Z^{\left(G\right)}$ branches lead to F(G) independent variables. Additionally, there is one more variable related to the central bead. Thus, the expression giving the number of independent variables of the *n*-th group n≤(G+1) reads:

(29) $$\begin{array}{c} F\left(n\right)=\left\{\begin{array}{cc} 1 & \text{for}\;n=1\\ F\left(n-1\right)+\sum_{i=1}^{n-1}F\left(i\right) & \text{for}\;G\geq n>1\\ F(G)+1 & \text{for}\;n=G+1 \end{array}\right. \end{array}$$

The recurrence Equation (29) can be solved (see Supplementary Materials), leading for 1≤n≤G to $F\left(n\right)=\frac{2}{\sqrt{5}}T_{2n-1}\left(\frac{\sqrt{5}}{2}\right)$, where $T_{i}\left(x\right)$ is the Chebyshev polynomial of the first kind [50]. Using a closed form representation of the Chebyshev polynomials [50], one obtains:

(30) $$\begin{array}{c} F\left(n\right)=\left\{\begin{array}{cc} \frac{1}{10}\left[\left(5-\sqrt{5}\right){\left(\frac{3+\sqrt{5}}{2}\right)}^{n}+\left(5+\sqrt{5}\right){\left(\frac{3-\sqrt{5}}{2}\right)}^{n}\right] & \text{for}\;G\geq n\geq 1\;\\ \frac{1}{10}\left[\left(5-\sqrt{5}\right){\left(\frac{3+\sqrt{5}}{2}\right)}^{G}+\left(5+\sqrt{5}\right){\left(\frac{3-\sqrt{5}}{2}\right)}^{G}\right]+1 & \text{for}\;n=G+1\; \end{array}\right. \end{array}$$

Now, we turn to the discussion of the degeneracy of the eigenmodes introduced above, which follows from the number of symmetric realizations with respect to the central bead of the T-fractal.

We start with the first group, which involves only the motion of terminal beads (i.e., beads of functionality 1). Let $N_{1}\left(G\right)$ be the total number of terminal beads at generation *G*. The construction of the T-fractal introduces a new terminal bead per bond of former generation; see Figure 1. The number of bonds at generation G-1 equals $3^{G-1}$; the number of terminal beads at generation G-1 is $N_{1}\left(G-1\right)$. Thus,

(31) $$\begin{array}{c} N_{1}\left(G\right)=3^{G-1}+N_{1}\left(G-1\right)=3^{G-1}+\cdots +3^{1}+3=\frac{3^{G}+3}{2} \end{array}$$

The first group involves only pairs of NNN terminal beads. These NNN terminal beads stem from terminal bonds of former generation. The number of such bonds is equal to the number of terminal beads. Therefore, the degeneracy of the eigenmodes of the first group for the T-fractal of generation *G*, $D_{1}\left(G\right)$, is given by:

(32) $$\begin{array}{c} D_{1}\left(G\right)=N_{1}\left(G-1\right)=\frac{3^{G-1}+3}{2} \end{array}$$

Furthermore, according to the iterative construction of a T-fractal, described in Section 1, each terminal bead leads to a $Z^{\left(2\right)}$ branch at the forthcoming iteration. Moreover, only the NNN terminal beads (i.e., those involved in the first group) result in two $Z^{\left(2\right)}$ branches that share the same gluing bead. Hence, $D_{1}\left(G\right)=D_{2}\left(G+1\right)=D_{3}\left(G+2\right)=...$, i.e., for the *n*-th group, we have:

(33) $$\begin{array}{c} D_{n}\left(G\right)=\frac{3^{G-n}+3}{2}\;\text{for}\;1\leq n\leq G \end{array}$$

For n=G, Equation (33) leads to $D_{G}\left(G\right)=2$ showing that for the *G*-th group, there are only two linearly independent realizations of two oppositely-moving $Z^{\left(G\right)}$ branches.

Since the central bead does not have any symmetric counterpart, the eigenmodes coming from the (G+1)-th group are nondegenerate. Summarizing, the degeneracies of the eigenmodes are given by:

(34) $$\begin{array}{c} D_{n}\left(G\right)=\left\{\begin{array}{cc} 1 & \text{for}\;n=G+1\\ \frac{3^{G-n}}{2}+\frac{3}{2} & \text{for}\;n\leq G \end{array}\right. \end{array}$$

The number of independent variables F(n), Equation (30), in combination with the degeneracy $D_{n}\left(G\right)$ for the corresponding group, Equation (34), gives the total number of eigenmodes N:

(35) $$\begin{array}{cc} N & =\sum_{n=1}^{G+1}F\left(n\right)\;D_{n}\left(G\right)=3^{G}+1=N\left(G\right) \end{array}$$

Equation (35) shows that N equals the number of beads of the corresponding T-fractal, i.e., the introduced set of hierarchical eigenmodes is a complete set of eigenvectors of $A^{\mathrm{STP}}$. The proof of Equation (35) is presented in the Supplementary Materials.

### 3.3. Reduced Matrices

Based on the groups of eigenvectors of the matrix $A^{\mathrm{STP}}$ (see Section 3.2), its eigenvalue spectrum can be determined using a set of matrices that are much smaller than $A^{\mathrm{STP}}$. The largest matrix of this set is a F(G+1)×F(G+1) matrix, whereas $A^{\mathrm{STP}}$ is a N×N matrix. Table 1 compares the values of *N* and F(G+1) for the first ten generations of T-fractals. Dealing with these matrices, it is necessary to distinguish between the matrices corresponding to the groups 1≤n≤G and the matrix corresponding to the (G+1)-th group of a T-fractal.

First, we consider the matrices of the first *G* groups. As discussed in Section 3.2, the description of the opposing movement of two $Z^{\left(n\right)}$ branches requires F(n) independent variables. The F(n)×F(n) matrix (which we call in the following the reduced matrix $A_{n}$) of equations of motion for these variables yields F(n) eigenvalues of the *n*-th group. In order to represent the reduced matrices $\left\{A_{n}\right\}$, one has to choose a numeration of the independent variables. A particular choice of the numeration does not play any role; our choice is presented in Supplementary Materials, see Figure S1.

Since one variable is sufficient to describe the opposing movement of two $Z^{\left(1\right)}$ branches, one obtains a single equation of motion (36). Hence, the corresponding coefficient matrix reads:

(36) $$A_{1}=\left(\mu_{1}-\rho \right)$$

The second group requires two variables to treat the opposing movement of two $Z^{\left(2\right)}$ branches. Consequently, the two equations of motion (37) lead to the coefficient matrix:

(37) $$A_{2}=\left(\begin{array}{cc} \mu_{1}+\rho & \nu_{1}\\ 2\nu_{1} & \mu_{3}-\rho \end{array}\right)$$

The equations of motion (38) of the third group result in the reduced matrix:

(38) $$A_{3}=\left(\begin{array}{ccccc} \mu_{1}+\rho & \nu_{1} & \rho & 0 & 0\\ 2\nu_{1} & \mu_{3}+\rho & \nu_{2} & 0 & \rho \\ 4\rho & 2\nu_{2} & \mu_{4} & \rho & \nu_{2}\\ 0 & 0 & \rho & \mu_{1} & \nu_{1}\\ 0 & 2\rho & \nu_{2} & \nu_{1} & \mu_{5}-\rho \end{array}\right)$$

The fourth group requires thirteen independent variables, whose dynamics is described through thirteen non-trivial equations of motion. The corresponding reduced matrix reads:

(39) $$A_{4}=\left(\begin{array}{ccccccccccccc} \mu_{1}+\rho & \nu_{1} & \rho & 0 & 0 & 0 & 0 & 0 & 0 & 0 & 0 & 0 & 0\\ 2\nu_{1} & \mu_{3}+\rho & \nu_{2} & 0 & \rho & 0 & 0 & 0 & 0 & 0 & 0 & 0 & 0\\ 4\rho & 2\nu_{2} & \mu_{4} & \rho & \nu_{2} & \rho & 0 & 0 & 0 & 0 & 0 & 0 & 0\\ 0 & 0 & \rho & \mu_{1} & \nu_{1} & \rho & 0 & 0 & 0 & 0 & 0 & 0 & 0\\ 0 & 2\rho & \nu_{2} & \nu_{1} & \mu_{5}+\rho & \nu_{2} & 0 & \rho & 0 & 0 & 0 & 0 & 0\\ 0 & 0 & 2\rho & 2\rho & 2\nu_{2} & \mu_{4} & \rho & \nu_{2} & 0 & 0 & \rho & 0 & 0\\ 0 & 0 & 0 & 0 & 0 & \rho & \mu_{1} & \nu_{1} & 0 & 0 & \rho & 0 & 0\\ 0 & 0 & 0 & 0 & 2\rho & \nu_{2} & \nu_{1} & \mu_{5} & 0 & \rho & \nu_{2} & 0 & \rho \\ 0 & 0 & 0 & 0 & 0 & 0 & 0 & 0 & \mu_{1}+\rho & \nu_{1} & \rho & 0 & 0\\ 0 & 0 & 0 & 0 & 0 & 0 & 0 & \rho & 2\nu_{1} & \mu_{3} & \nu_{2} & 0 & \rho \\ 0 & 0 & 0 & 0 & 0 & \rho & \rho & \nu_{2} & 2\rho & \nu_{2} & \mu_{4} & \rho & \nu_{2}\\ 0 & 0 & 0 & 0 & 0 & 0 & 0 & 0 & 0 & 0 & \rho & \mu_{1} & \nu_{1}\\ 0 & 0 & 0 & 0 & 0 & 0 & 0 & \rho & 0 & \rho & \nu_{2} & \nu_{1} & \mu_{5}-\rho \end{array}\right)$$

From the fifth group on, an iterative construction of the reduced matrices, based on the construction of the eigenmodes presented in Section 3.2, is possible. Figure 5 illustrates that a branch $Z^{\left(n\right)}$ can be decomposed into two terminal $Z^{\left(n-1\right)}$ branches and one internal ${\tilde{Z}}^{\left(n-1\right)}$ branch whose starting bead *s* coincides with the starting bead of the whole branch $Z^{\left(n\right)}$. The corresponding F(n)×F(n) reduced matrix $A_{n}$ describing opposing movements of two $Z^{\left(n\right)}$ branches has the following form:

(40) $$\begin{array}{c} A_{n}=\left(\begin{array}{cc} {\tilde{A}}_{n-1} & W_{12}\\ W_{21} & {\tilde{L}}_{n-1} \end{array}\right) \end{array}$$

In Equation (40), the F(n-1)×F(n-1) matrix ${\tilde{A}}_{n-1}$ describes the two terminal $Z^{\left(n-1\right)}$ branches, whereas the internal ${\tilde{Z}}^{\left(n-1\right)}$ branch is described by the (F(n)-F(n-1))×(F(n)-F(n-1)) matrix ${\tilde{L}}_{n-1}$. The blocks $W_{12}$ and $W_{21}$ reflect the interaction of the two external branches with the internal branch. The exact form of all of these matrices is presented in Appendix A.

The reduced F(G+1)×F(G+1) matrix arising from the (G+1)-th group is denoted by $B_{G}$. In the (G+1)-th group, the beads symmetric with respect to the central bead have the same amplitude. With the help of Figure 1 and Figure 2, one can easily construct the matrices of the first two generations of T-fractals,

(41) $$\begin{array}{c} B_{1}=\left(\begin{array}{cc} \mu_{1}+2\rho & \nu_{1}\\ 3\nu_{1} & \mu_{2} \end{array}\right) \end{array}$$

and:

(42) $$\begin{array}{c} B_{2}=\left(\begin{array}{ccc} \mu_{1}+\rho & \nu_{1} & \rho \\ 2\nu_{1} & \mu_{3}+2\rho & \nu_{2}\\ 6\rho & 3\nu_{2} & \mu_{4} \end{array}\right) \end{array}$$

The reduced matrices $B_{G}$ for T-fractals of generation G≥3 can be constructed based on the matrix $A_{G}$:

(43) $$\begin{array}{c} B_{G}=\left(\begin{array}{cc} {\bar{A}}_{G} & C_{12}\\ C_{21} & \mu_{4} \end{array}\right) \end{array}$$

The reason for the structure of Equation (43) is as follows: The central bead of the T-fractal can move only in the (G+1)-th group. For G≥2, it is an internal bead connected to three other internal NN beads. In the case of such a configuration, according to Equation (16), the last diagonal element of $B_{G}$ (which represents the central bead) is given by $\mu_{4}$. The matrix $A_{G}$ describes opposing movements of two $Z^{\left(G\right)}$ branches. Considering the (G+ 1)-th group, one observes uniform movements of three $Z^{\left(G\right)}$ branches. In this way, $A_{G}$ can be used to construct ${\bar{A}}_{G}$:

(44) $$\begin{array}{c} {\left({\bar{A}}_{G}\right)}_{ij}={\left(A_{G}\right)}_{ij}+3\rho \delta_{i,c}\delta_{c,j} \end{array}$$

where c=F(G) numerates the variable related to beads, which are NN to the central bead.

The central bead of the T-fractal interacts with the three $Z^{\left(G\right)}$ branches via NN and NNN interactions. The corresponding F(G)×1 matrix $C_{12}$ and 1×F(G) matrix $C_{21}$ are given by:

(45) $$\begin{array}{c} {\left(C_{12}\right)}_{ij}=\delta_{1,j}\left[\rho \delta_{i,c-2}+\rho \delta_{i,c-1}+\nu_{2}\delta_{i,c}\right] \end{array}$$

and:

(46) $$\begin{array}{c} {\left(C_{21}\right)}_{ij}=\delta_{i,1}\left[3\rho \delta_{c-2,j}+3\rho \delta_{c-1,j}+3\nu_{2}\delta_{c,j}\right] \end{array}$$

respectively.

### 3.4. Eigenvalue Spectra of $A^{\mathrm{STP}}$

**Figure 7:**
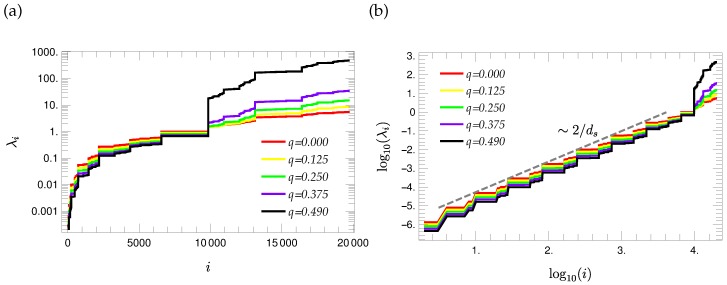
Eigenvalue spectra of G=9 T-fractals plotted in semi-logarithmic (**a**) and in double logarithmic (**b**) scales for different values of the stiffness parameter *q*.

The determination of the eigenvalue spectra is performed based on the procedure introduced in Section 3.3. Figure 7 presents the eigenvalue spectra corresponding to ninth generation T-fractals for different choices of the stiffness parameter *q*. The eigenvalues are presented in ascending order in semi-logarithmic scales since the largest and smallest non-vanishing eigenvalue differ strongly. It turns out that the eigenvalue spectra have a stair-like shape. Moreover, one observes a plateau in the middle region of the spectrum, whose width is independent of the choice of the stiffness parameter. This plateau is determined by the eigenvalue with the largest degeneracy, i.e., by the eigenvalue of the first group $\lambda^{\left(1\right)}$. The spectra displayed in Figure 7 depend qualitatively on the stiffness parameter *q*. $\lambda^{\left(1\right)}$, and all smaller eigenvalues get smaller with increasing *q*, whereas the larger eigenvalues get larger.

**Figure 8:**
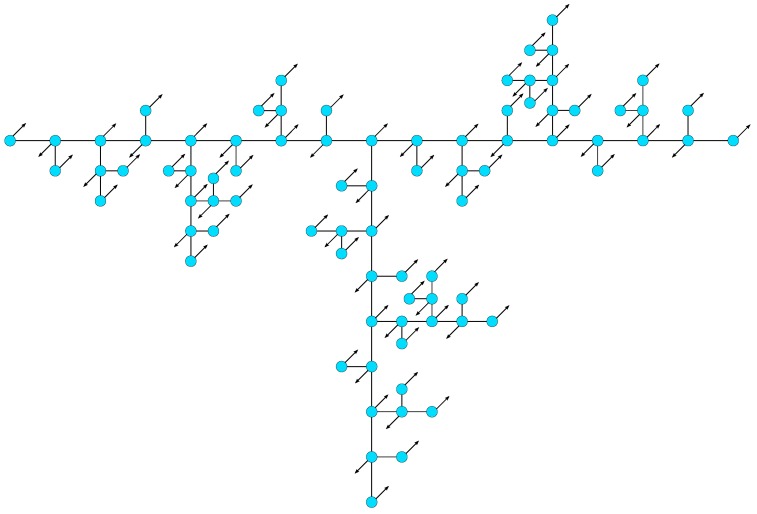
Schematic representation of the eigenmode corresponding to the largest eigenvalue of a G=4 T-fractal.

One can understand this characteristic behavior by looking at the corresponding relaxation times $\tau_{k}$, which are related to the eigenvalues through [27]:

(47) $$\begin{array}{c} \tau_{k}=\frac{\tau_{0}}{\lambda_{k}}\;\text{with}\;\tau_{0}=\frac{\zeta }{K} \end{array}$$

and at the corresponding eigenmodes. A numerical analysis of the eigenmodes belonging to large eigenvalues shows that a large part of adjacent groups of beads that are described by the same independent variable moves oppositely, but with the same amplitude. Such a movement of the beads allows a fast relaxation of the corresponding eigenmode. The more beads are moving in opposite directions, the faster the relaxation takes place. Since the number of moving beads increases with growing group number, the largest occurring eigenvalue belongs to an eigenmode of the (G+1)-th group, which is proven by the numerical analysis. The relaxation times of eigenmodes that involve mainly the motion of adjacent beads in alternating directions decrease with increasing stiffness parameter *q*, so that the associated eigenvalues increase. Figure 8 illustrates the eigenmode corresponding to the largest eigenvalue of a T-fractal of generation G=4.

The analysis of the eigenvectors corresponding to eigenvalues less than or equal to $\lambda^{\left(1\right)}$ yields that large domains of the macromolecule move with the same phase. In the limiting case, all beads of the fractal move with the same amplitude in the same direction. This translational eigenmode corresponds to the eigenvalue λ=0. An increase of the stiffness parameter enlarges the size of the macromolecule, so that the relaxation times of eigenmodes related to collective motions of large domains increase, i.e., the corresponding eigenvalues decrease.

The analysis of the eigenvalue spectrum plotted in double logarithmic scales (Figure 7b) shows that the steps corresponding to eigenvalues smaller than $\lambda^{\left(1\right)}$ follow a straight line. The approximate slope of this straight line is related to the spectral dimension of the T-fractal [21] $d_{s}=\frac{\log(9)}{\log(6)}$ by the quotient $\frac{2}{d_{s}}=\frac{2\log(6)}{\log(9)}\approx 1.63$, i.e.,

(48) $$\begin{array}{c} \lambda_{i}∼i^{\frac{2}{d_{s}}} \end{array}$$

for eigenvalues $\lambda_{i}$ smaller than $\lambda^{\left(1\right)}$. We note that this scaling holds for all considered values of the stiffness parameter *q*. Thus, the scaling exponent is robust under the introduction of local constraints. This behavior is in line with the scaling of the spectral density n(λ), for which then:

(49) $$\begin{array}{c} n\left(\lambda \right)∼\frac{di(\lambda )}{d\lambda }∼\lambda^{d_{s}/2-1} \end{array}$$

holds, in accordance with the definition of the spectral dimension $d_{s}$ [51].

**Figure 9:**
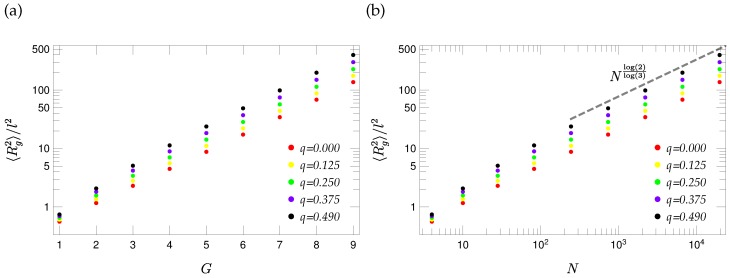
Gyration radius of T-fractals for different values of the stiffness parameter *q* plotted in (**a**) as a function of generation *G* and in (**b**) as a function of number of beads *N*.

Based on the eigenvalue spectra $\left\{\lambda_{i}\right\}$, one can readily calculate the gyration radius $\langle R_{g}^{2}\rangle$ [52],

(50) $$\begin{array}{c} \langle R_{g}^{2}\rangle =\frac{\ell^{2}}{N}\sum_{i=2}^{N}\frac{1}{\lambda_{i}} \end{array}$$

where the sum runs over all eigenvalues, except $\lambda_{1}=0$ associated with the translational motion. Figure 9 shows $\langle R_{g}^{2}\rangle /\ell^{2}$ for different values of the stiffness parameter *q*. As can be inferred from the figure, $\langle R_{g}^{2}\rangle$ grows with increasing *q*, because of the importance of small eigenvalues that decrease with growing *q*; see Equation (50). Moreover, as is typical for fractals, $\langle R_{g}^{2}\rangle$ shows a scaling for large molecular weights. Indeed, using Equation (49), one obtains [53] $\langle R_{g}^{2}\rangle ∼N^{\frac{2-d_{s}}{d_{s}}}=N^{\frac{\log2}{\log3}}$, as can be observed in Figure 9b.

### 3.5. Mechanical Relaxation

**Figure 10:**
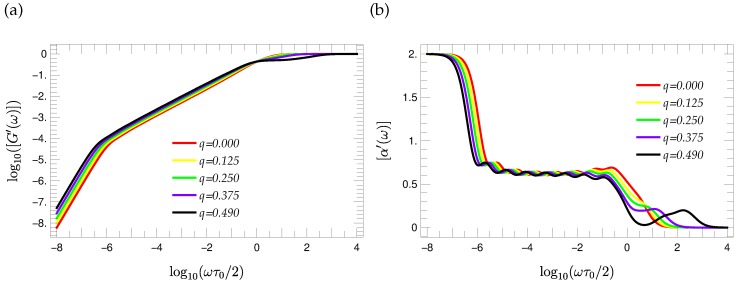
(**a**) Reduced storage moduli $\left[G^{\prime }\left(\omega \right)\right]$ of G=9 T-fractals and (**b**) the corresponding local slopes of the $\left[G^{\prime }\left(\omega \right)\right]$ curves for different values of stiffness parameter *q*.

**Figure 11:**
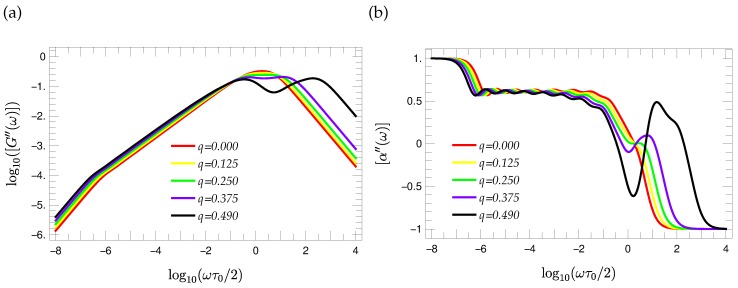
(**a**) Reduced loss moduli $\left[G^{\prime \prime }\left(\omega \right)\right]$ of G=9 T-fractals and (**b**) the corresponding local slopes of the $\left[G^{\prime \prime }\left(\omega \right)\right]$ curves for different values of stiffness parameter *q*.

The knowledge of the eigenvalue spectra allows the calculation of many dynamical characteristics [27]. Here, we focus on the reduced storage and loss moduli; see Equations (10) and (11) of Section 2. Figure 10 and Figure 11 show the reduced storage and loss moduli of a T-fractal of generation G=9 for different choices of the stiffness parameter *q*, respectively. The comparison of Figure 10 and Figure 11 shows that the stiffness has a stronger influence on the curve shape of the loss modulus than on the storage modulus. Therefore, we consider first the loss modulus.

Obviously, the increasing value of the stiffness parameter *q* leads to a broadening of the $\left[G^{\prime \prime }\left(\omega \right)\right]$ curves that is accompanied by the development of a local minimum. The significance of this minimum increases with the increasing of the stiffness parameter. The eigenvalue spectra helps to understand this behavior of the curve. The increasing of the stiffness parameter leads to a larger step height between the most degenerated eigenvalue $\lambda^{\left(1\right)}$ and larger eigenvalues, so that a pseudo gap arises in the eigenvalue spectrum. As a consequence of the enlarging gap that is caused by the increasing of the stiffness parameter, the maxima of contributions to $\left[G^{\prime \prime }\left(\omega \right)\right]$ corresponding to eigenvalues that are larger than $\lambda^{\left(1\right)}$ are shifted to higher frequencies, so that a local minimum arises in the range of middle frequencies of the loss modulus. Such a local minimum is present for semiflexible dendrimers [39] and less pronounced for semiflexible Vicsek fractals [40]. We note that the modes of the first two groups for T-fractals have exactly the same pattern (although different multiplicity) as for dendrimers [39,43], whereas for Vicsek fractals, only the first group of eigenmodes resembles that of the dendrimers [40]. These two groups lead to most degenerate eigenvalues (see Equation (34)), and hence, they play a major role for the position of the two maxima and the local minima.

On the other hand, one observes a scaling behavior in the range of middle frequencies; such a feature is typical for fractals and not for dendrimers. For the purpose of a more precise analysis, the derivation:

(51) $$\begin{array}{c} \left[\alpha^{\prime \prime }\left(\omega \right)\right]=\frac{d}{d\log_{10}\left(\omega \right)}\log_{10}\left[G^{\prime \prime }\left(\omega \right)\right] \end{array}$$

representing the local slope of $\left[G^{\prime \prime }\left(\omega \right)\right]$, is utilized. The curve shape of $\left[\alpha^{\prime \prime }\left(\omega \right)\right]$ for T-fractals of generation G=9 is depicted in Figure 11b. Obviously, there is a wavy pattern of the $\left[\alpha^{\prime \prime }\left(\omega \right)\right]$ functions in the region of middle frequencies. However, the corresponding oscillations are independent of the choice of the stiffness parameter being between the values 0.55 and 0.65. The approximately uniform oscillation justifies the determination of mean values; one finds $\bar{\left[\alpha^{\prime \prime }\right]}\approx 0.615,0.615,0.613,0.610,0.604$ for q=0,0.125,0.250,0.375,0.490. Hence the slope of the loss modulus is independent of *q* and proportional to $\omega^{\bar{\left[\alpha^{\prime \prime }\right]}}\approx \omega^{0.61}$ in the range of middle frequencies, in line with general expectations [27,54,55] $\left[G^{\prime \prime }\left(\omega \right)\right]∼\omega^{\frac{d_{s}}{2}}$. We note, that $\left[G^{\prime }\left(\omega \right)\right]$ shows the same scaling behavior in the range of middle frequencies, if one considers the derivative:

(52) $$\begin{array}{c} \left[\alpha^{\prime }\left(\omega \right)\right]=\frac{d}{d\log_{10}\left(\omega \right)}\log_{10}\left[G^{\prime }\left(\omega \right)\right] \end{array}$$

depicted in Figure 10b. Comparing $\left[\alpha^{\prime }\left(\omega \right)\right]$ and $\left[\alpha^{\prime \prime }\left(\omega \right)\right]$, one observes the same wavy pattern. Moreover, inspection of the $\left[\alpha^{\prime }\left(\omega \right)\right]$ and $\left[\alpha^{\prime \prime }\left(\omega \right)\right]$ curves for T-fractals of other generations (not shown here) indicates that the number of appearing local maxima in the wavy region is equal to (G-2).

## 4. Conclusions

In this paper, we have studied the dynamics of hyperbranched, dendritic macromolecules modeled through T-fractals. The symmetry of the T-fractal structure enabled us to construct a full set of eigenmodes and to analyze it in detail. Moreover, the set has reduced the computational efforts by having much smaller reduced dynamical matrices. The analysis of the corresponding eigenvalue spectra has shown a significant broadening of the spectra with increasing stiffness. Thus, the relaxation of the large-scale eigenmodes feels the increase of size of the macromolecule with growing stiffness and becomes slower; meanwhile, the relaxation of the small scale eigenmodes becomes faster due to the locally-constrained motion. These features become relevant for the mechanical relaxation moduli, which show a broadening with increasing stiffness. Moreover, while for high frequencies, the moduli reflect the local dendritic nature of the T-fractals (this behavior is more pronounced by the loss modulus for higher stiffness), a broad range of intermediate frequencies reveals through a scaling the fractal character of the macromolecules (which is less influenced by stiffness), a feature that is rather typical for hyperbranched polymers [56].

## Figures and Tables

**Table 1:** Comparison of *N* and F(G+1) for the first ten generations of T-fractals.

*G*	1	2	3	4	5	6	7	8	9	10
*N*	4	10	28	82	244	730	2188	6562	19,684	59,050
F(G+1)	2	3	6	14	35	90	234	611	1598	4182

**Figure A1:**
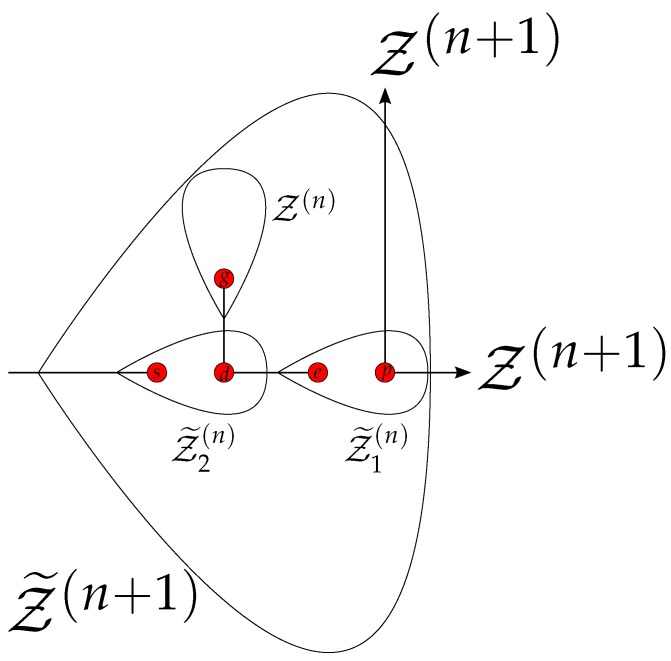
Composition of an internal ${\tilde{Z}}^{\left(n+1\right)}$ branch.

## Supplementary Materials

The following are available online at www.mdpi.com/2073-4360/8/7/263/s1. Proof of Equations (30) and (34). Figure S1. Numeration of eigenvectors’ amplitudes used for the (**a**) first, (**b**) second, (**c**) third and (**d**) fourth groups. The gluing beads colored by black are immobile.

## Abbreviations

The following abbreviations are used in this manuscript:

GGS: generalized Gaussian structures
STP: semiflexible treelike polymers
NN: nearest neighbor
NNN: next-nearest neighbor

## Appendix A. Iterative Procedure for the Calculation of the Matrix A_*n*+1_ from A_*n*_

Here, we present an iterative procedure for the calculation of the matrix $A_{n+1}$ from $A_{n}$ introduced in Section 3.3. According to Equation (40):

(A1) $$\begin{array}{c} A_{n+1}=\left(\begin{array}{cc} {\tilde{A}}_{n} & W_{12}\\ W_{21} & {\tilde{L}}_{n} \end{array}\right) \end{array}$$

where the numeration of the beads is chosen in a such way that the last diagonal element of ${\tilde{A}}_{n}$ and of ${\tilde{L}}_{n}$ represents the starting beads *k* of the terminal branches Z and the starting bead *s* of the internal branch $\tilde{Z}$, respectively; see Figure 5 for notations and the Supplementary Materials for the details of the numeration procedure. Hence, the starting beads of the two terminal branches Z are given by the diagonal element ${\left({\tilde{A}}_{n}\right)}_{kk}$ with k=F(n). Further, the bead *p* of $\tilde{Z}$ connecting the two Z branches that is marked in Figure 5 corresponds to the first diagonal element of ${\tilde{L}}_{n}$.

${\tilde{A}}_{n}$ and $A_{n}$ differ from each other in the last diagonal element *k*, since $A_{n}$ describes the opposing movement of two $Z^{\left(n\right)}$ branches, whereas ${\tilde{A}}_{n}$ represents the uniform movement of two such branches. Therefore, one can use $A_{n}$ to construct ${\tilde{A}}_{n}$:

(A2) $$\begin{array}{c} {\left({\tilde{A}}_{n}\right)}_{ij}={\left(A_{n}\right)}_{ij}+2\rho \delta_{i,k}\delta_{k,j} \end{array}$$

The starting beads *k* of the two terminal Z branches interact by NN interaction with the bead *p* and by NNN interaction with the NN of *p* in $\tilde{Z}$. In addition to that, there is an NNN interaction between the NNs of the beads *k*, belonging to the respective Z branch and the bead *p*. Hence, one obtains the interaction matrix:

(A3) $$\begin{array}{c} {\left(W_{12}\right)}_{ij}=\delta_{1,j}\left[\rho \delta_{i,k-2}+\rho \delta_{i,k-1}+\nu_{2}\delta_{i,k}\right]+\rho \delta_{i,k}\delta_{3,j} \end{array}$$

The other off-diagonal block $W_{21}$ reflects the interaction of *p*, and its NN beads in $\tilde{Z}$ with the two beads *k* and their NN beads in Z. These interactions result in:

(A4) $$\begin{array}{c} {\left(W_{21}\right)}_{ij}=\delta_{i,1}\left[2\rho \delta_{k-2,j}+2\rho \delta_{k-1,j}+2\nu_{2}\delta_{k,j}\right]+2\rho \delta_{i,3}\delta_{k,j} \end{array}$$

In order to determine the matrix ${\tilde{L}}_{n}$, a more precise consideration of the internal branch $\tilde{Z}$ is necessary. Figure A1 illustrates that an internal ${\tilde{Z}}^{\left(n+1\right)}$ branch consists of two internal branches ${\tilde{Z}}_{1}^{\left(n\right)}$ and ${\tilde{Z}}_{2}^{\left(n\right)}$, as well as of one terminal $Z^{\left(n\right)}$ branch. Hereby, the ${\tilde{Z}}_{2}^{\left(n\right)}$ branch contains the starting bead *s* of the ${\tilde{Z}}^{\left(n+1\right)}$ branch. The bead *p*, connecting the two terminal $Z^{\left(n+1\right)}$, branches belongs to the ${\tilde{Z}}_{1}^{\left(n\right)}$ branch.

Since an iterative construction of the internal ${\tilde{Z}}^{\left(n\right)}$ branch is possible, also the corresponding matrix ${\tilde{L}}_{n}$ can be constructed in an iterative way:

(A5) $$\begin{array}{c} {\tilde{L}}_{n+1}=\left(\begin{array}{ccc} {\hat{L}}_{n} & {\tilde{W}}_{12} & {\tilde{W}}_{13}\\ {\tilde{W}}_{21} & {\hat{A}}_{n} & {\tilde{W}}_{23}\\ {\tilde{W}}_{31} & {\tilde{W}}_{32} & {\tilde{L}}_{n} \end{array}\right) \end{array}$$

where ${\tilde{L}}_{n}$ is a (2F(n)-F(n-1))×(2F(n)-F(n-1)) matrix. The three block matrices ${\hat{L}}_{n}$, ${\tilde{A}}_{n}$ and ${\tilde{L}}_{n}$ represent the branches ${\tilde{Z}}_{1}^{\left(n\right)}$, $Z^{\left(n\right)}$ and ${\tilde{Z}}_{2}^{\left(n\right)}$, respectively. ${\hat{L}}_{n}$ and ${\tilde{L}}_{n}$ are l×l matrices, with l=2F(n)-F(n-1).

The starting beads *e* and *s* of ${\tilde{Z}}_{1}^{\left(n\right)}$ and ${\tilde{Z}}_{2}^{\left(n\right)}$ correspond to the diagonal elements ${\left({\hat{L}}_{n}\right)}_{ll}$ and ${\left({\tilde{L}}_{n}\right)}_{ll}$, respectively. The bead *d* that is connected to the beads *g* and *e* corresponds to the first diagonal element of ${\tilde{L}}_{n}$. The remaining matrix ${\hat{A}}_{n}$ is a m×m matrix, with m=F(n), whose last diagonal element represents the starting bead *g* of $Z^{\left(n\right)}$. The matrices ${\hat{L}}_{n}$ and ${\tilde{L}}_{n}$ differ from each other by the last diagonal element *l*, since the bead *s* of the ${\tilde{Z}}_{2}^{\left(n\right)}$ branch is in NN position to an identical bead, whereas this is not the case considering the starting bead *e* of the ${\tilde{Z}}_{1}^{\left(n\right)}$ branch. Hence, the relation between the two matrices is given by:

(A6) $$\begin{array}{c} {\left({\hat{L}}_{n}\right)}_{ij}={\left({\tilde{L}}_{n}\right)}_{ij}+\rho \delta_{i,l}\delta_{l,j} \end{array}$$

Furthermore, the $Z^{\left(n\right)}$ branch does not have an identical counterpart sharing the same gluing bead, so that the last diagonal element of the m×m matrix $A_{n}$ has to be modified in order to obtain ${\hat{A}}_{n}$:

(A7) $$\begin{array}{c} {\left({\hat{A}}_{n}\right)}_{ij}={\left(A_{n}\right)}_{i,j}+\rho \delta_{i,m}\delta_{m,j} \end{array}$$

The l×m matrix ${\tilde{W}}_{12}$ and m×l matrix ${\tilde{W}}_{21}$ describe the NNN interactions of the beads *e* and *g* belonging to the branches ${\tilde{Z}}_{1}^{\left(n\right)}$ and $Z^{\left(n\right)}$. They are given by:

(A8) $$\begin{array}{c} {\left({\tilde{W}}_{12}\right)}_{ij}=\rho \delta_{i,l}\delta_{m,j} \end{array}$$

and:

(A9) $$\begin{array}{c} {\left({\tilde{W}}_{21}\right)}_{ij}=\rho \delta_{i,m}\delta_{l,j} \end{array}$$

Four beads are involved in the interaction between the branches ${\tilde{Z}}_{1}^{\left(n\right)}$ and ${\tilde{Z}}_{2}^{\left(n\right)}$, namely the bead *e*, its NN in ${\tilde{Z}}_{1}^{\left(n\right)}$, the bead *d* and its NN in ${\tilde{Z}}_{2}^{\left(n\right)}$. The corresponding interaction matrices are denoted as ${\tilde{W}}_{13}$ and ${\tilde{W}}_{31}$:

(A10) $$\begin{array}{c} {\left({\tilde{W}}_{13}\right)}_{ij}=\delta_{1,j}\left[\rho \delta_{i,l-2}+\rho \delta_{i,l-1}+\nu_{2}\delta_{i,l}\right]+\rho \delta_{i,l}\delta_{3,j} \end{array}$$

and:

(A11) $$\begin{array}{c} {\left({\tilde{W}}_{31}\right)}_{ij}=\delta_{i,1}\left[\rho \delta_{l-2,j}+\rho \delta_{l-1,j}+\nu_{2}\delta_{l,j}\right]+\rho \delta_{i,3}\delta_{l,j} \end{array}$$

Finally, one has to handle the interaction of the branches $Z^{\left(n\right)}$ and ${\tilde{Z}}^{\left(n\right)}$. The four beads taking part in this interaction are *g* and *d* and their NN belonging to the respective branches. The interaction of these beads yields the two matrices:

(A12) $$\begin{array}{c} {\left({\tilde{W}}_{23}\right)}_{ij}=\delta_{1,j}\left[\rho \delta_{i,m-2}+\rho \delta_{i,m-1}+\nu_{2}\delta_{i,m}\right]+\rho \delta_{i,m}\delta_{3,j} \end{array}$$

and:

(A13) $$\begin{array}{c} {\left({\tilde{W}}_{31}\right)}_{ij}=\delta_{i,1}\left[\rho \delta_{m-2,j}+\rho \delta_{m-1,j}+\nu_{2}\delta_{m,j}\right]+\rho \delta_{i,3}\delta_{m,j} \end{array}$$

In order to be able to construct $A_{5}$ using the iterative procedure of Equation (40), the knowledge of ${\tilde{L}}_{4}$ is necessary. This requires the specific knowledge of ${\tilde{L}}_{3}$ that can be taken from Equation (39), whose last 8×8 diagonal block reads:

(A14) $${\tilde{L}}_{3}=\left(\begin{array}{cccccccc} \mu_{4} & \rho & \nu_{2} & 0 & 0 & \rho & 0 & 0\\ \rho & \mu_{1} & \nu_{1} & 0 & 0 & \rho & 0 & 0\\ \nu_{2} & \nu_{1} & \mu_{5} & 0 & \rho & \nu_{2} & 0 & \rho \\ 0 & 0 & 0 & \mu_{1}+\rho & \nu_{1} & \rho & 0 & 0\\ 0 & 0 & \rho & 2\nu_{1} & \mu_{3} & \nu_{2} & 0 & \rho \\ \rho & \rho & \nu_{2} & 2\rho & \nu_{2} & \mu_{4} & \rho & \nu_{2}\\ 0 & 0 & 0 & 0 & 0 & \rho & \mu_{1} & \nu_{1}\\ 0 & 0 & \rho & 0 & \rho & \nu_{2} & \nu_{1} & \mu_{5}-\rho \end{array}\right)$$

## References

[1] Gao C., Yan D. (2004). Hyperbranched polymers: From synthesis to applications. Prog. Polym. Sci..

[2] Voit B.I., Lederer A. (2009). Hyperbranched and highly branched polymer architectures: Synthetic strategies and major characterization aspects. Chem. Rev..

[3] Yan D., Gao C., Frey H. (2011). Hyperbranched Polymers: Synthesis, Properties, and Applications.

[4] Lederer A., Burchard W. (2015). Hyperbranched Polymers: Macromolecules in between Deterministic Linear Chains and Dendrimer Structures.

[5] Fischer M., Vögtle F. (1999). Dendrimers: From design to application—A progress report. Angew. Chem. Int. Ed..

[6] Ballauff M., Likos C.N. (2004). Dendrimers in solution: Insight from theory and simulation. Angew. Chem. Int. Ed..

[7] Sowinska M., Urbanczyk-Lipkowska Z. (2014). Advances in the chemistry of dendrimers. New J. Chem..

[8] Lederer A., Burchard W., Khalyavina A., Lindner P., Schweins R. (2013). Is the universal law valid for branched polymers?. Angew. Chem. Int. Ed..

[9] Hölter D., Burgath A., Frey H. (1997). Degree of branching in hyperbranched polymers. Acta Polym..

[10] Lyulin A.V., Adolf D.B., Davies G.R. (2001). Computer simulations of hyperbranched polymers in shear flows. Macromolecules.

[11] Sheridan P.F., Adolf D.B., Lyulin A.V., Neelov I., Davies G.R. (2002). Computer simulations of hyperbranched polymers: The influence of the Wiener index on the intrinsic viscosity and radius of gyration. J. Chem. Phys..

[12] Polińska P., Gillig C., Wittmer J.P., Baschnagel J. (2014). Hyperbranched polymer stars with Gaussian chain statistics revisited. Eur. Phys. J. E.

[13] Jurjiu A., Dockhorn R., Mironova O., Sommer J.U. (2014). Two universality classes for random hyperbranched polymers. Soft Matter.

[14] Wawrzyńska E., Sikorski A., Zifferer G. (2015). Monte Carlo simulation studies of regular and irregular dendritic polymers. Macromol. Theory Simul..

[15] Lederer A., Burchard W., Hartmann T., Haataja J.S., Houbenov N., Janke A., Friedel P., Schweins R., Lindner P. (2015). Dendronized hyperbranched macromolecules: Soft matter with a novel type of segmental distribution. Angew. Chem. Int. Ed..

[16] Kahng B., Redner S. (1989). Scaling of the first-passage time and the survival probability on exact and quasi-exact self-similar structures. J. Phys. A Math. Gen..

[17] Matan O., Havlin S. (1989). Mean first-passage time on loopless aggregates. Phys. Rev. A.

[18] Maritan A., Sartoni G., Stella A.L. (1993). Singular dynamical renormalization group and biased diffusion on fractals. Phys. Rev. Lett..

[19] Burioni R., Cassi D., Regina S. (1999). Cutting-decimation renormalization for diffusive and vibrational dynamics on fractals. Phys. A.

[20] Burioni R., Cassi D., Corberi F., Vezzani A. (2007). Phase-ordering kinetics on graphs. Phys. Rev. E.

[21] Agliari E. (2008). Exact mean first-passage time on the T-graph. Phys. Rev. E.

[22] Haynes C.P., Roberts A.P. (2008). Global first-passage times of fractal lattices. Phys. Rev. E.

[23] Zhang Z., Lin Y., Zhou S., Wu B., Guan J. (2009). Mean first-passage time for random walks on the T-graph. New J. Phys..

[24] Lin Y., Wu B., Zhang Z. (2010). Determining mean first-passage time on a class of treelike regular fractals. Phys. Rev. E.

[25] Agliari E., Blumen A., Mülken O. (2010). Quantum-walk approach to searching on fractal structures. Phys. Rev. A.

[26] Dolgushev M., Guérin T., Blumen A., Bénichou O., Voituriez R. (2015). Contact kinetics in fractal macromolecules. Phys. Rev. Lett..

[27] Gurtovenko A., Blumen A. (2005). Generalized Gaussian Structures: Models for polymer systems with complex topologies. Polymer Analysis Polymer Theory.

[28] Rouse P.E. (1953). A theory of the linear viscoelastic properties of dilute solutions of coiling polymers. J. Chem. Phys..

[29] Markelov D.A., Dolgushev M., Gotlib Y.Y., Blumen A. (2014). NMR relaxation of the orientation of single segments in semiflexible dendrimers. J. Chem. Phys..

[30] Markelov D.A., Falkovich S.G., Neelov I.M., Ilyash M.Y., Matveev V.V., Lähderanta E., Ingman P., Darinskii A.A. (2015). Molecular dynamics simulation of spin–lattice NMR relaxation in poly-l-lysine dendrimers: Manifestation of the semiflexibility effect. Phys. Chem. Chem. Phys..

[31] Bixon M., Zwanzig R. (1978). Optimized Rouse–Zimm theory for stiff polymers. J.Chem. Phys..

[32] Guenza M., Perico A. (1992). A reduced description of the local dynamics of star polymers. Macromolecules.

[33] La Ferla R. (1997). Conformations and dynamics of dendrimers and cascade macromolecules. J. Chem. Phys..

[34] Von Ferber C., Blumen A. (2002). Dynamics of dendrimers and of randomly built branched polymers. J. Chem. Phys..

[35] Dolgushev M., Blumen A. (2009). Dynamics of semiflexible treelike polymeric networks. J. Chem. Phys..

[36] Dolgushev M., Blumen A. (2009). Dynamics of semiflexible chains, stars, and dendrimers. Macromolecules.

[37] Kumar A., Biswas P. (2010). Dynamics of semiflexible dendrimers in dilute solutions. Macromolecules.

[38] Kumar A., Rai G.J., Biswas P. (2013). Conformation and intramolecular relaxation dynamics of semiflexible randomly hyperbranched polymers. J. Chem. Phys..

[39] Fürstenberg F., Dolgushev M., Blumen A. (2012). Analytical model for the dynamics of semiflexible dendritic polymers. J. Chem. Phys..

[40] Fürstenberg F., Dolgushev M., Blumen A. (2013). Dynamics of semiflexible regular hyperbranched polymers. J. Chem. Phys..

[41] Qi Y., Dolgushev M., Zhang Z. (2014). Dynamics of semiflexible recursive small-world polymer networks. Sci. Rep..

[42] Galiceanu M., Reis A.S., Dolgushev M. (2014). Dynamics of semiflexible scale-free polymer networks. J. Chem. Phys..

[43] Cai C., Chen Z.Y. (1997). Rouse dynamics of a dendrimer Model in the *ϑ* Condition. Macromolecules.

[44] Winkler R.G., Reineker P., Harnau L. (1994). Models and equilibrium properties of stiff molecular chains. J. Chem. Phys..

[45] Mansfield M.L., Stockmayer W.H. (1980). Unperturbed dimensions of wormlike stars. Macromolecules.

[46] Doi M. (1996). Introduction to Polymer Physics.

[47] Gurtovenko A.A., Gotlib Y.Y., Blumen A. (2002). Rouse dynamics of polymer networks bearing dendritic wedges. Macromolecules.

[48] Gurtovenko A.A., Markelov D.A., Gotlib Y.Y., Blumen A. (2003). Dynamics of dendrimer-based polymer networks. J. Chem. Phys..

[49] Koda S. (2015). Equivalence between a generalized dendritic network and a set of one-dimensional networks as a ground of linear dynamics. J. Chem. Phys..

[50] Mason J.C., Handscomb D.C. (2003). Chebyshev Polynomials.

[51] Alexander S., Orbach R. (1982). Density of states on fractals: «fractons». J. Phys. Lett..

[52] Dolgushev M., Berezovska G., Blumen A. (2010). Cospectral polymers: Differentiation via semiflexibility. J. Chem. Phys..

[53] Sommer J.-U., Blumen A. (1995). On the statistics of generalized Gaussian structures: Collapse and random external fields. J. Phys. A.

[54] Friedrich C. (1991). Relaxation and retardation functions of the Maxwell model with fractional derivatives. Rheol. Acta.

[55] Schiessel H., Blumen A. (1995). Mesoscopic pictures of the sol-gel transition: Ladder models and fractal networks. Macromolecules.

[56] Sokolov I.M., Klafter J., Blumen A. (2002). Fractional kinetics. Phys. Today.

