# Supplementary material for: Relaxation Dynamics of Semiflexible Fractal Macromolecules
Source: Polymers (Basel). 2016 Jul 15;8(7):263. doi: 10.3390/polym8070263 (PMC6432473; doi:10.3390/polym8070263)
Supplement: Supplementary file 1 [file polymers-08-00263-s001.pdf]

# Supplementary Materials: Relaxation Dynamics of Semiflexible Fractal Macromolecules

**Jonas Mielke and Maxim Dolgushev**

## 1. Supplementary Proofs

### 1.1. Number of Independent Variables

As discussed in Section 3.2, the number of independent variables corresponding to the $n$-th group can be recursively expressed as:

$$F(n) = \begin{cases} 1 & \text{for } n = 1 \\ F(n-1) + \sum_{i=0}^{n-1} F(i) & \text{for } G \geq n > 1 \\ F(G) + 1 & \text{for } n = G + 1 \end{cases} \quad \text{(S1)}$$

Let us rewrite the recursive Equation (S1) as:

$$F(n) = F(n-1) + \sum_{i=1}^{n-1} F(i) = 2F(n-1) + \sum_{i=1}^{n-2} F(i) \quad \text{(S2)}$$

Rewriting Equation (S2) for the case $n-1$ leads to:

$$F(n-1) - F(n-2) = \sum_{i=1}^{n-2} F(i) \quad \text{(S3)}$$

Insertion of Equations (S3) in (S2) yields:

$$F(n) = 3F(n-1) - F(n-2) \quad \text{(S4)}$$

Equation (S4) is the recursive relation for Chebyshev polynomials of the third kind [50]:

$$\mathrm{V}_n(x) = 2x\mathrm{V}_{n-1}(x) - \mathrm{V}_{n-2}(x) \quad \text{for } n = 2, 3, \ldots \quad \text{(S5)}$$

with the starting values $\mathrm{V}_0(x) = 1$ and $\mathrm{V}_1(x) = 2x - 1$ for $x = 3/2$. Using the relation between Chebyshev polynomials of the third kind $\mathrm{V}_n(x)$ and of the first kind $\mathrm{T}_n(x)$ [50],

$$\mathrm{V}_n(x) = \frac{\mathrm{T}_{2n+1}(u)}{u} \quad \text{(S6)}$$

where $u = \sqrt{(1+x)/2}$, we obtain:

$$F(n) = \frac{2\mathrm{T}_{2n-1}(\frac{\sqrt{5}}{2})}{\sqrt{5}} \quad \text{(S7)}$$

Moreover, Chebyshev polynomials of the first kind can be rewritten in closed analytical form [50],

$$\mathrm{T}_n(x) = \frac{1}{2}\left[(x+\sqrt{x^2-1})^n + (x-\sqrt{x^2-1})^n\right] \tag{S8}$$

With this expression, Equation (S1) transforms finally to:

$$F(n) = \begin{cases} \frac{1}{10}\left[(5-\sqrt{5})\left(\frac{3+\sqrt{5}}{2}\right)^n + (5+\sqrt{5})\left(\frac{3-\sqrt{5}}{2}\right)^n\right] & \text{for } G \geq n \geq 1 \\ \frac{1}{10}\left[(5-\sqrt{5})\left(\frac{3+\sqrt{5}}{2}\right)^G + (5+\sqrt{5})\left(\frac{3-\sqrt{5}}{2}\right)^G\right] + 1 & \text{for } n = G+1 \end{cases} \tag{S9}$$

### 1.2. Completeness of Set of Eigenmodes

The number of eigenmodes of the $n$-th group $(n = 1, ..., G+1)$ is related to the number of eigenvalues $F(n)$ and the corresponding degeneracy $D_n(G)$ by the product $F(n)D_n(G)$. Hence, the total number of eigenmodes for a T-fractal of generation $G$ is given by:

$$\mathcal{N} = \sum_{n=1}^{G+1} F(n)D_n(G) = S_1 + S_2 + S_3 + S_4 + \frac{1}{10}\left[(5-\sqrt{5})\left(\frac{3+\sqrt{5}}{2}\right)^G + (5+\sqrt{5})\left(\frac{3-\sqrt{5}}{2}\right)^G\right] + 1 \tag{S10}$$

where:

$$S1 \equiv \frac{1}{10}\sum_{n=1}^{G}\left[(5-\sqrt{5})\left(\frac{3+\sqrt{5}}{2}\right)^n\right]\frac{3^{G-n}}{2} = \frac{2^{-G-1}}{5}(-5-2\sqrt{5})\left((3+\sqrt{5})^G - 6^G\right) \tag{S11}$$

$$S2 \equiv \frac{1}{10}\sum_{n=1}^{G}\left[(5+\sqrt{5})\left(\frac{3-\sqrt{5}}{2}\right)^n\right]\frac{3^{G-n}}{2} = 2^{-G-3}\left(-4+\frac{8}{\sqrt{5}}\right)\left((3-\sqrt{5})^G - 6^G\right) \tag{S12}$$

$$S3 \equiv \frac{1}{10}\sum_{n=1}^{G}\left[(5-\sqrt{5})\left(\frac{3+\sqrt{5}}{2}\right)^n\right]\frac{3}{2} = 3\cdot 2^{-G-1}\frac{1}{\sqrt{5}}\left((3+\sqrt{5})^G - 2^G\right) \tag{S13}$$

$$S4 \equiv \frac{1}{10}\sum_{n=1}^{G}\left[(5+\sqrt{5})\left(\frac{3-\sqrt{5}}{2}\right)^n\right]\frac{3}{2} = 3\cdot 2^{-G-1}\frac{1}{\sqrt{5}}\left(2^G - (3-\sqrt{5})^G\right) \tag{S14}$$

Combining Equations (S10) to (S14) yields:

$$\mathcal{N} = 3^G + 1 \tag{S15}$$

## 2. Supplementary Figure: Numeration of Groups

Here, we exemplify the numeration used for the set of equations of motion and the corresponding coefficient matrix,; see Figure S1 for groups $n = 1$ to 4. In general, we choose the numeration that starts from the periphery. According to the structure of eigenmodes, beads of one branch that are arranged in a symmetric way with respect to the gluing bead move with the same amplitude, and the counterparts of the second branch move with an amplitude of the same absolute value in the opposite direction. Thus, all beads, whose movement amplitude has the same absolute value, are numbered by the same number.

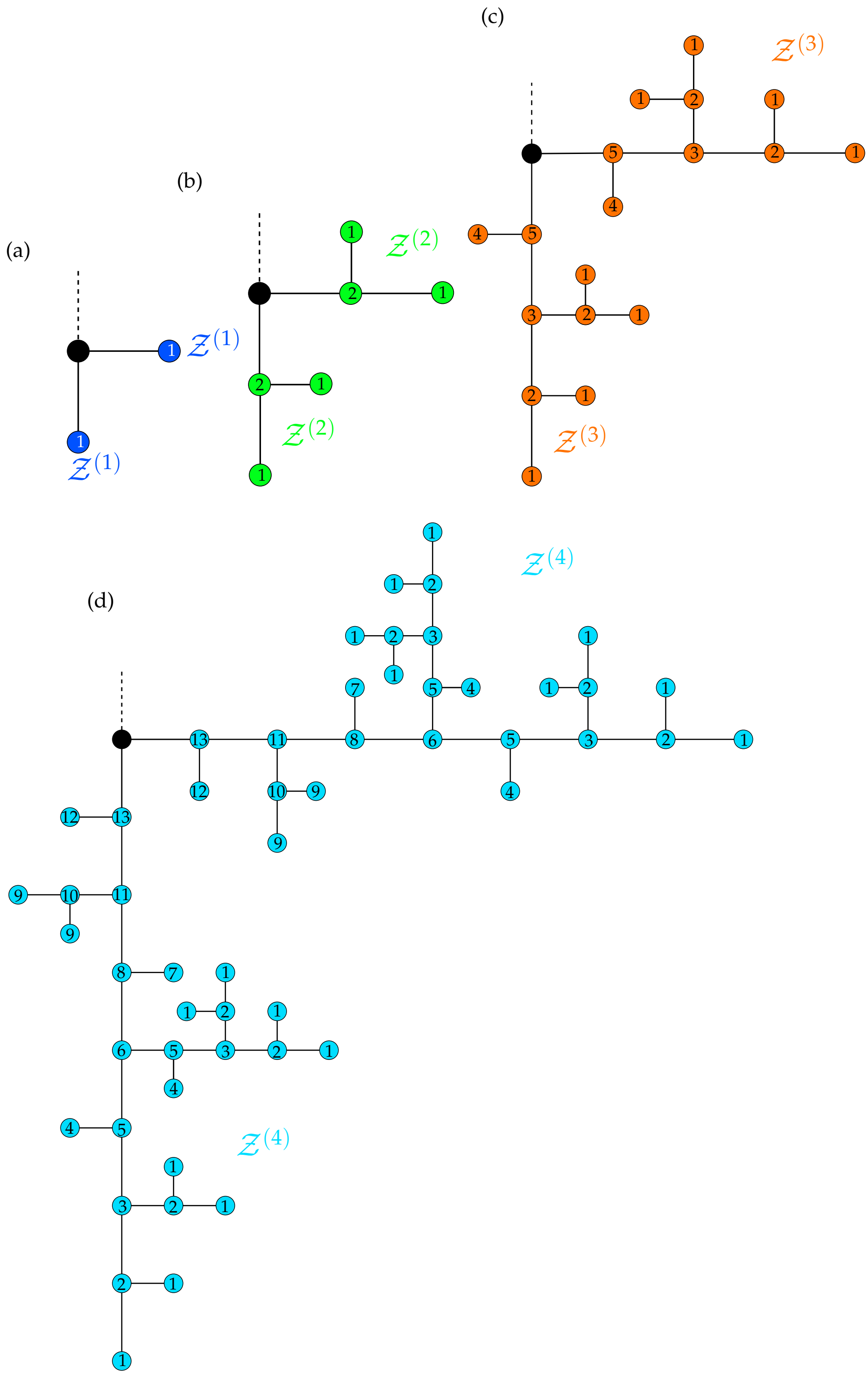


**Figure S1.** Numeration of eigenvectors' amplitudes used for the (**a**) first, (**b**) second, (**c**) third and (**d**) fourth groups. The gluing beads colored by black are immobile.
